# Levels and prognostic impact of circulating markers of inflammation, endothelial activation and extracellular matrix remodelling in patients with lung cancer and chronic obstructive pulmonary disease

**DOI:** 10.1186/s12885-018-4659-0

**Published:** 2018-07-13

**Authors:** Janna Berg, Ann Rita Halvorsen, May-Bente Bengtson, Kristin A. Taskén, Gunhild M. Mælandsmo, Arne Yndestad, Bente Halvorsen, Odd Terje Brustugun, Pål Aukrust, Thor Ueland, Åslaug Helland

**Affiliations:** 1 Department of Cancer Genetics, Institute for Cancer Research, Radium Hospital, Oslo University Hospital, Oslo, Norway; 2 Department of Medicine, Vestfold Hospital Trust, Tønsberg, Norway; 3 Department of Tumour Biology, Institute for Cancer Research, Radium Hospital, Oslo University Hospital, Oslo, Norway; 4 Research Institute of Internal Medicine, Oslo University Hospital, Rikshospitalet, Oslo, Norway; 5 Institute of Clinical Medicine, University of Oslo, Oslo, Norway; 6 Section of Oncology, Drammen Hospital, Vestre Viken Hospital Trust, Drammen, Norway

**Keywords:** COPD, Lung cancer, Serum markers, Inflammation, Prognosis, Protein

## Abstract

**Background:**

The development of both chronic obstructive pulmonary disease (COPD) and lung cancer (LC) is influenced by smoking related chronic pulmonary inflammation caused by an excessive innate immune response to smoke exposure. In addition, the smoking induced formation of covalent bonds between the carcinogens and DNA and the accumulation of permanent somatic mutations in critical genes are important in the carcinogenic processes, and can also induce inflammatory responses.

How chronic inflammation is mirrored by serum markers in COPD and LC and if these markers reflect prognosis in patients with LC is, however, largely unknown.

**Methods:**

Serum levels of 18 markers reflecting inflammation, endothelial activation and extracellular matrix remodelling were analysed in 207 patients with non-small lung carcinoma (NSCLC) before surgery and 42 COPD patients. 56% of the LC patients also suffered from COPD. The serum samples were analysed by enzyme immunoassays.

**Results:**

Serum levels of OPG, PTX3, AXL, ALCAM, sCD163, CD147, CatS and DLL1 were significantly higher in patients with COPD as compared to patients with LC. High sTNFR1 levels were associated with improved progression free survival (PFS) and overall survival (OS) in LC patients with (PFS hazard ratio (HR) 0.49, OS HR 0.33) and without COPD (OS HR 0.30). High levels of OPG were associated with improved PFS (HR 0.17) and OS (HR 0.14) for LC with COPD. CRP was significantly associated with overall survival regardless of COPD status.

**Conclusion:**

Several markers reflecting inflammation, endothelial activation and extracellular matrix remodelling are elevated in serum from patients with COPD compared to LC patients. Presence of COPD might influence the levels of circulating biomarkers. Some of these markers are also associated with prognosis.

**Electronic supplementary material:**

The online version of this article (10.1186/s12885-018-4659-0) contains supplementary material, which is available to authorized users.

## Background

Lung cancer (LC) is the second most common type of cancer in men and women and the most common cause of cancer-related death [1]. Prognosis depends heavily on stage of disease and approximately 70% of LC patients are diagnosed with locally advanced or metastatic disease, beyond curative potential [2]. Hence, LC screening is investigated worldwide as a means to increase early diagnosis of LC. In the National Lung Screening Trial in the US, LC screening of heavy smokers has proven to reduce LC mortality [3]. However, computed tomography (CT) screening for LC unfortunately has a high rate of false positive findings (96.4%) limited by being anatomic in nature, unable to differentiate between benign and neoplastic lesions. Several research projects have aimed to identify biomarkers that can supplement CT when screening for LC, in order to reduce the number of false positives. Such studies could potentially also give insight into pathogenic mechanisms in LC, which in long-term could potentially improve the therapeutic options.

Despite many publications on LC screening biomarkers, none has yet been established in clinical practice [4–8]. Serum proteins associated with LC have been identified, but the findings are generally based on comparisons of LC patients versus healthy subjects, comprising a very different control group than the LC high risk group eligible for screening. LC is up to five times more likely to occur in smokers with airflow obstruction than in those with normal lung function, and both chronic obstructive pulmonary disease (COPD) and LC are associated with smoking behaviour [9]. Tobacco smoking is carcinogenic and known to induce the formation of DNA-adducts and mutations. The innate immune system is activated, and inflammation is induced, and this mechanism has been shown to be important in the carcinogenic process [10].

Chronic pulmonary inflammation with increased levels of neutrophils, macrophages and bronchial epithelial cells releasing cytokines, including Tumour Necrosis Factor alpha (TNF), ALCAM and osteoproterin lead to secretion of acute phase proteins (e.g. CRP, PTX3) from the liver, further worsening inflammation [10–14].

Axl is a known proto-oncogene associated with epithelial-to-mesenchymal transition (EMT), higher metastatic potential, therapeutic resistance, and overall worse prognosis, and studies have shown that PTX3, AXL og ALCAM are associated with metastatic lung cancer [11, 12].

The aim of this study is to identify differences in levels of the selected serum markers in patients with non-small cell lung carcinoma (NSCLC) and patients with COPD. The serum markers were selected based on their ability to reflect inflammation, endothelial cell activation and extracellular matrix (ECM) remodelling, processes that are involved in the pathogenesis of both LC and COPD. In addition, we wanted to elucidate if some of these could give prognostic information in relation to LC progression and prognosis. The design is a case control study, including patients with NSCLC and patients with COPD.

## Methods

The aim of this study is to identify differences in the selected serum markers in patients with NSCLC and patients with COPD. In addition, the prognostic impact of the serum markers is investigated. The design is a case control study, including patients with NSCLC and patients with COPD.

### Study population

#### Lung cancer group

207 patients with operable NSCLC, surgically treated at Rikshospitalet, Oslo University Hospital, Oslo, Norway between May 2007 and June 2012 were included. Clinical characteristics of the NSCLC patients were collected from hospital medical records. Tumours were staged in accordance with the Union for International Cancer Control, Tumour, Node, Metastasis 7 (TNM 7). Most of the patients had stage I (approx. 57%) and stage II (approx. 26%) (Table 1). 55.6% of the LC group had COPD and the majority had moderate COPD. We grouped the LC patients with moderate, severe and very severe COPD in one group (LC with COPD) and the LC patients with mild or no COPD in another group (LC^only^).

**Table 1:** Clinical characteristics of lung cancer and COPD patients

	Lung cancer without COPD	Lung cancer with COPD	COPD	*p*-value
Number of patients (%)	92 (44.4%)	115 (55.6%)	42	
Age on randomization, y				
median	67.5	65.8	70	*p* = 0.175
< 59	23 (25%)	21 (18.3%)	6 (14.3%)	
60–69	35 (38%)	58 (50.4%)	14 (33.3%)	
70+	34 (37%)	36 (31.3%)	22 (52.4%)	
Sex				
Female	41 (45%)	52 (45%)	23 (55%)	*p* = 0.237
Male	51 (55%)	63 (55%)	19 (45%)	
COPD 2017 classification				
II Moderate (FEV1 50–80%)		99 (86.1%)	16 (38%)	*p* < 0.001
III Severe (FEV1 30–50%)		15 (13%)	18 (43%)	
IV Very severe (FEV1 < 30%)		1 (0.9%)	8 (19%)	
Steroids				
Oral	No info	No info	4 (9.5%)	
Inhalation	No info	No info	27 (64.3%)	
No steroids			11(26.2%)	
Smoking status				
Never	9 (9.8%)	1 (0.9%)	3 (7%)	*p* = 0.016
Former	57 (62%)	65 (56.5%)	29 (69%)	
Current	26 (28.2%)	49 (42.6%)	10 (24%)	
Pack-years smoked among former/current smokers	83	114	39	*p* = 0.008
< 30	47 (57%)	44 (39%)	11 (29%)	
30–39	16 (20%)	28 (25%)	10 (26%)	
40–49	4 (5%)	20 (18%)	11 (29%)	
50+	15 (18%)	22 (19%)	6 (16%)	
No information	1		1	
Never smokers	9	1	3	
Lung cancer histology				
Squamous cell carcinoma	35 (38%)	69 (60%)		*p* = 0.001
Adenocarcinoma	57 (62%)	46 (40%)		
Lung cancer stage				
I	46 (50%)	72 (62.6%)		*p* = 0.105
II	28 (30%)	25 (21.7%)		
III	16 (17%)	17 (14.8%)		
IV	2 (3%)	1 (0.9%)		
5-year survival	54 (59%)	68 (59%)		

Clinical characteristics. *FEV* forced expiratory volume

#### COPD group

Serum samples from 50 patients with COPD stadium II-IV were obtained at the Department of Medicine, Vestfold Hospital Trust, Tønsberg, Norway (COPD^only^). Clinical information was acquired from hospital records (Table 1). All COPD patients included were in a regular follow-up and had no signs of LC or other forms of cancer prior to blood sampling. The patients were also followed for a minimum of 2 years after blood sampling with no sign of cancer. COPD was diagnosed according to the criteria of the Global Initiative for Chronic Obstructive Lung Disease (GOLD)(http://goldcopd.org). Subjects with a history of asthma, other pulmonary disease or serious heart disease were excluded (*n* = 8), leaving 42 patients in the analyses. A smoking history of more than 10 pack-years or significant occupational exposure for asbestos or other industrial dust was required for inclusion of the COPD patients.

### Spirometry

Spirometry was performed according to the American Thoracic Society/European Respiratory Society guidelines. Lung function was measured by spirometry using the Jaeger Master Lab (Eric Jaeger, Wurzburg, Germany) with subjects in the sitting position, and the highest value of forced expiratory volume in 1 s (FEV1) and forced vital capacity (FVC) from at least two technically satisfactory manoeuvres differing by less than 5% was recorded, as well as the ratio FEV1/FVC. Predicted values were obtained from Quanjer et al. [13]. The subjects had to avoid the use of short-acting β2-agonists at least 8 h prior to the test.

### Blood sample processing

Blood samples from NSCLC patients were collected before surgery. Blood was collected in SST ™serum tubes (BD Biosciences), kept in room temperature appending coagulation and then processed at 2450 g for 12 min within 1 h after sampling. Finally, the samples were transferred in 250 μl aliquots into cryogenic vials and stored at − 80 °C until usage. Serum samples in the COPD cohort were pre-processed under strictly defined and equal conditions as the samples obtained from the NSCLC patients, stored at the same site as the NSCLC samples and processed further at the same centre by the same personnel.

### Serum analyses

Serum levels of 18 markers of inflammation and fibrosis, selected based on previous association with LC and its prognosis (Table 2) were measured in duplicate using commercially available reagents by enzyme- immunoassay (EIA; all proteins except vWF: R&D Systems, Minneapolis, MN, USA; vWF: DakoCytomation, Glostrup, Denmark) in a 384-format using the combination of a SELMA (Jena, Germany) pipetting robot and a BioTek (Winooski, VT, USA) dispenser/washer (EL406). Primary and secondary antibody concentrations were used according to manufacturer (Coating 1–4 μg/mL; secondary 0.2–2 μg/mL). Assay volume was 25 μL and coating was performed in phosphate buffered saline (PBS, SCBT, Heidelberg, Germany). Subsequent assay buffer was 1% bovine serum albumin (VWR, Radnor, PA, USA) in PBS while sample diluent was assay buffer with 25% heat inactivated fetal calf serum (Gibco, Thermo Fisher Scientific, Waltman, MA, USA). Wash buffer was PBS with 0.05% tween20 and 3 wash cycles were included per step. Samples were incubated overnight at 4 °C. Absorption was read at 450 nm with wavelength correction set to 540 nm using an EIA plate reader (Synergy H1 Hybrid, Biotek, Vinooski, VT, USA). Intra- and inter-assay coefficients of variation were < 10% for all ass.

**Table 2:** Markers included in the study

Markers for	Protein short name	Protein full name
Vascular inflammation	OPG	Osteoprotegrin
	PTX3	Pentraxin 3
	Axl	Tyrosine-protein kinase receptor
	CXCL16	C-X-C motif chemokine ligand 16
	vWF	Von Willebrand factor
	ePCR	Endothelial cell protein C receptor
Activated monocytes/	Alcam (CD166)	Activated leukocyte cell adhesion molecule
macrophage markers	PARC	p53-associated parkin-like cytoplasmic protein
	sCD163	Cluster of differentiation 163
	CD14	Cluster of differentiation 14
	Gal3BP	Galectin-3-binding protein
Extracellular matrix	CD147	Cluster of differentiation 147
remodeling (ECM) and fibrosis		(Basigin. EMMPRIN)
	Endostatin	
	GDF15	Growth differentiation factor-15
	Cats	Cathepsin S (Chloramphenicol acetyl transferase)
General inflammation	sTNFR1	Tumour necrosis factor receptor 1
	CRP	C-reactive protein
Notch	DLL1	Delta-like protein 1

### Statistical analyses

Data are reported using descriptive statistics with percentages, means, medians and ranges. Differences in log transformed protein serum levels between the clinical groups were calculated using one-way ANOVA test. For multiple comparison (compare means between COPD, LC with and without COPD groups) Tukey HSD (Honestly Significant Difference) was applied. Prior to analysis the data was inspected for normal distribution by using histogram and for equal variance distribution using Levene’s test. Pearson correlation was applied to check if the proteins correlated with covariates such as age, sex and pack-years. To control for these variables, we conducted a multivariate analysis of covariance (MANCOVA). Bonferroni adjustment was applied to correct for multiple analyses. Overall survival and progression free survival was analysed using multivariate cox regression analysis. Factors such as stage, age, gender, histology and pack-years were included as covariates. Data were analysed using the SPSS software package version 21 (SPSS, Chicago, IL, USA). Two-sided *P*-values < 0.05 were considered statistical significant.

**Fig. 1:**
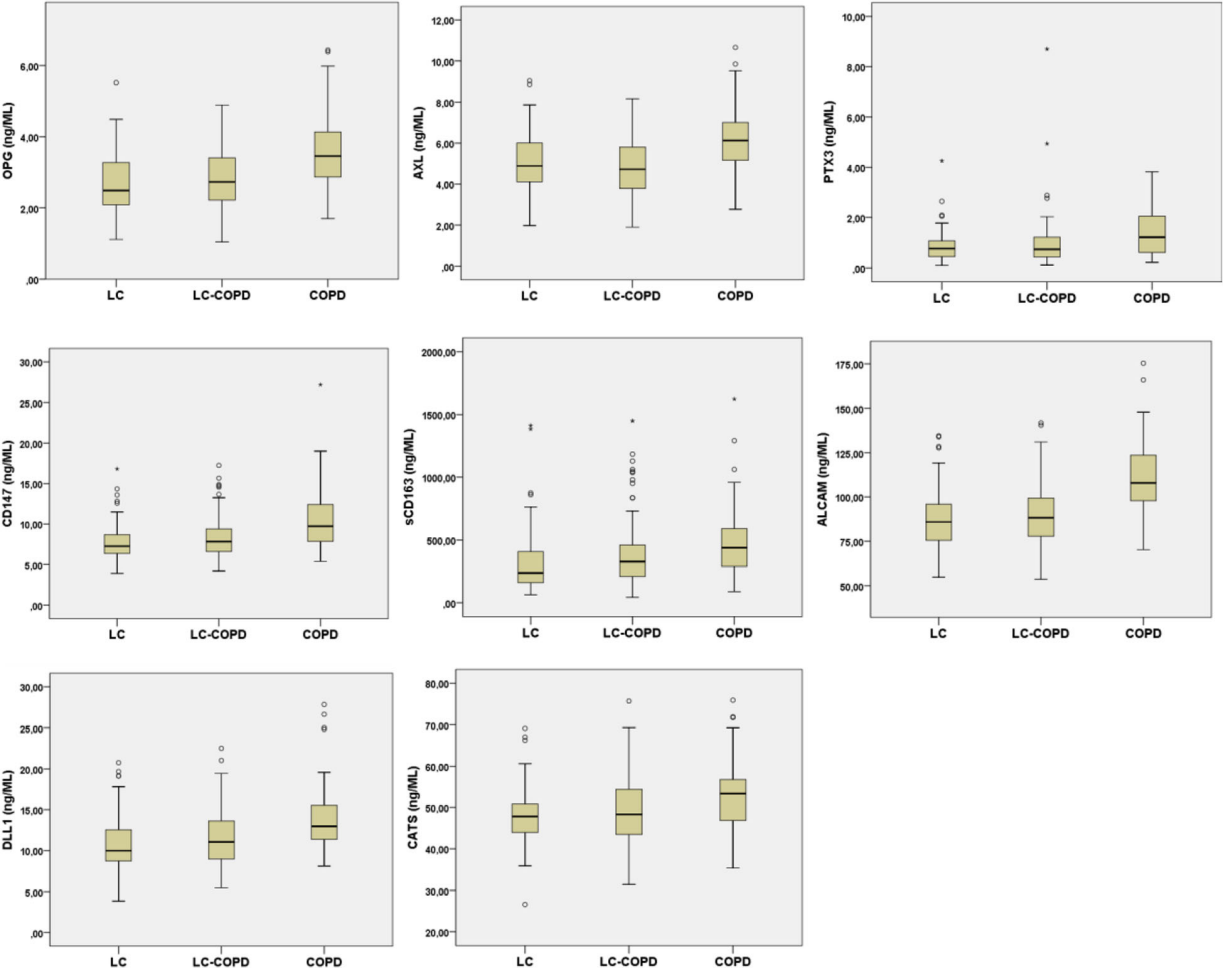
Serum levels of the proteins significantly differentially expressed in patients with COPD compared to patients with lung cancer. Legends: LC = Patients with Lung cancer, LC-COPD = Patients with both Lung cancer and COPD, COPD = Patients with only COPD

Comparisons are made as illustrated in Fig. 1.

## Results

### Characteristics of lung cancer patients and COPD patients

Serum samples from 207 LC patients and 42 patients with COPD were analysed for 18 markers reflecting inflammation, endothelial cell activation and ECM remodelling (Table 2). There was no significant difference in median age and gender between the different groups, but COPD patients had more severe COPD than LC patients with COPD (*p* < 0.001). There were more current smokers in the group of LC patients with COPD (43%) than in LC patients without COPD (28%) and COPD patients without cancer (24%). Almost 10% of LC patients without COPD had never smoked. Patients with cancer and COPD had a significantly higher median number of pack years (Table 1).

A significantly higher number of patients with squamous carcinoma had COPD compared to adenocarcinoma (*p* = 0.001). Log-rank test did not show different overall survival in patients with adenocarcinoma and squamous carcinoma (Overall survival *p* = 0.91 and Progression free survival *p* = 0.99). Five-year overall survival for the LC patients was 59% (Table 1).

### Serum protein levels in lung cancer patients and COPD patients

**Fig. 2:**
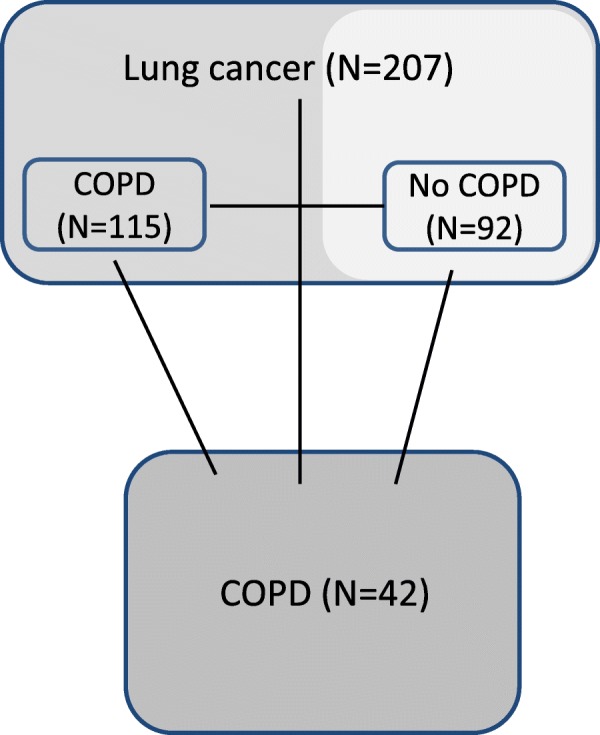
The material in this study and the different comparisons. COPD = Chronic obstructive pulmonary disease

Levels of OPG, PTX3, AXL, ALCAM, sCD163, CD147, CatS and DLL1 were significantly higher in serum from COPD patients compared to the complete LC patient-cohort regardless of COPD, after multivariate analyses of covariance (corrected for age, gender and pack-years) and correction for multiple testing. None of the proteins were significantly more abundant in serum from the patients with LC with and without COPD than in the COPD patients (Table 3, Fig. 2).

**Table 3:** Serum protein levels in lung cancer patients and COPD patients**.** LC^COPD^ –lung cancer with COPD. LC^only^ –lung cancer with and without COPD

MANCOVA			MANCOVA		ANOVA		
Protein	COPD (n = 42) vs LC (*n* = 207)	BC-sign	COPD / LC^only^ / LC^copd^	BC-sign	COPD vs LC^copd^	COPD vs LC^only^	LC^copd^ vs LC^only^
OPG	*p* < 0.001	Sign.	*p* < 0.001	Sign.	*p* < 0.001	*p* < 0.001	ns
PTX3	*p* = 0.001	Sign.	*p* = 0.002	Sign.	*p* = 0.04	*p* = 0.001	ns
Axl	*p* < 0.001	Sign.	*p* < 0.001	Sign.	*p* < 0.001	*p* = 0.002	ns
CXCL16	*p* = 0.019	ns	*p* = 0.005	ns	ns	*p* = 0.002	*p* = 0.023
Alcam (CD166)	*p* < 0.001	Sign.	p < 0.001	Sign.	*p* < 0.001	*p* < 0.001	ns
sCD163	*p* = 0.001	Sign.	*p* = 0.001	Sign.	ns	*p* = 0.01	ns
CD147	*p* < 0.001	Sign.	*p* < 0.001	Sign.	*p* < 0.001	*p* < 0.001	ns
Endostatin	ns	ns	*p* = 0.005	ns	ns	ns	*p* = 0.033
Cats	*p* = 0.002	Sign.	*p* = 0.004	ns	*p* = 0.029	*p* = 0.004	ns
CRP	ns	ns	*p* = 0.004	ns	*p* = 0.018	ns	*p* = 0.001
DLL1	*p* < 0.001	Sign.	*p* < 0.001	Sign.	*p* < 0.001	*p* < 0.001	ns

ANOVA and MANCOVA analyses comparing circulating protein levels in patients with COPD and patients with lung cancer with and without COPD. One-way MANCOVA was performed on two groups (COPD and Lung cancer with and without COPD), and on three groups (COPD, lung cancer with COPD and lung cancer without COPD). One-way ANOVA was conducted to explore the differences in the levels of proteins between the three groups; COPD (n = 42), lung cancer with COPD (*n* = 116) and lung cancer without COPD (n = 92). Multiple testing is controlled for by Bonferroni correction. Only significant markers are shown

One-way ANOVA was conducted to explore differences in serum markers between the three groups; patients with COPD (COPD^only^, *n* = 42), patients with LC with COPD (*n* = 115) and LC without COPD (*n* = 92). Post hoc comparison using Tukey’s test identified 11 markers with significantly different levels between the three groups (Table 3, ANOVA). After correction for covariance and multiple testing, we found significantly higher serum levels of OPG, PTX3, AXL, DLL1, CD147 and ALCAM in COPD patients compared to LC patients with and without COPD. sCD163 was significantly higher in COPD patients compared to LC patients without COPD. Levels of three proteins (CXCL16, endostatin and CRP) were significantly elevated in LC patients with COPD versus LC patients without COPD in the ANOVA analysis. This was not significant after adjusting for multiple testing (Table 3, MANCOVA). Figure 2 illustrates level differences in 8 biomarkers (OPG, PTX3, AXL, ALCAM, sCD163, CD147, Cats and DLL1) between patients with COPD, LC with and without COPD and LC with COPD. Correlation between serum markers and steroid use in the COPD group.

We found a negative correlation between steroid use (both systemic and inhalation), and serum levels of CXCL16 and CD147 (*r* = − 0.375, *p* = 0.014 and *r* = − 0.359, *p* = 0.020), and a positive correlation between soluble CD14 levels and steroid use (*r* = 0.419, *p* = 0.006).

### Prognostic significance

High levels of both sTNFR1 and OPG were significantly associated with improved survival, OPG with both progression free and overall survival among LC patients with COPD, and sTNFR1 with both progression free and overall survival among all LC patients with and without COPD and the LC patients with COPD (Table 4). In contrast to the “beneficial” associations of high OPG and sTNFR1 levels, higher levels of CRP were associated with decreased overall survival in our cohort irrespective of COPD status. More modest associations with outcome were found for PTX3, ePCR, PARC and vWF, with high levels of PTX3 and PARC associated with worse progression free survival (LC and COPD and total LC, respectively), and high ePCR associated with better overall survival in patients with LC without COPD. Finally, levels of OPG and vWF were significantly associated with progression free and overall survival in squamous carcinoma. In adenocarcinomas, level of sTNFR1 and CRP had a significant impact on progression free survival (Table 4).

**Table 4:** Survival analyses. LC^tot^ - lung cancer total - lung cancer with and without COPD LC^COPD^ –lung cancer with COPD. LC –lung cancer with and without COPD

	PFS			OS			PFS histology		OS histology	
Protein	LC ^tot^	LC	LC^COPD^	LC ^tot^	LC	LC^COPD^	SQCC	AC	SQCC	AC
OPG	ns	ns	p = 0.01 HR = 0.17 [0.04–0.70]	ns	ns	p = 0.03 HR = 0.14 [0.03–0.8]	p = 0.02 HR = 0.19 [0.05–0.75]	ns	*p* = 0.03 HR = 0.16 [0.03–0.81]	ns
PTX3	ns	ns	p = 0.01 HR = 1.70 [1.11–2.61]	ns	ns	ns	ns	ns	ns	ns
vWF	ns	ns	ns	ns	ns	ns	*p* = 0.05 HR = 3.14 [1.01–9.78]	ns	p = 0.01 HR = 7.68 [1.69–34.77]	ns
ePCR	ns	ns	ns	ns	p = 0.01 HR = 0.29 [0.11–0.77]	ns	ns	ns	ns	ns
PARC	p = 0.01 HR = 1.93 [1.16–3.22]	ns	ns	ns	ns	ns	ns	ns	ns	ns
Cats	ns	ns	ns	ns	ns	ns	ns	ns	ns	p = 0.01 HR = 39.44 [2.27–684.2]
sTNFR1	p = 0.03 HR = 0.57 [0.35–0.94]	ns	p = 0.03 HR = 0.49 [0.25–0.93]	p = 0.01 HR = 0.45 [0.25–0.83]	p = 0.04 HR = 0.3 [0.09–0.95]	*p* = 0.01 HR = 0.33 [0.15–0.73]	ns	p = 0.01 HR = 0.37 [0.18–0.78]	p = 0.02 HR = 0.31 [0.11–0.85]	p = 0.001 HR = 0.24 [0.10–0.58]
CRP	ns	ns	ns	p = 0.002 HR = 1.41 [1.13–1.75]	p = 0.01 HR = 1.73 [1.14–2.63]	p = 0.17 HR = 1.59 [1.09–2.33]	ns	p = 0.03 HR = 1.39 [1.04–1.85]	p = 0.02 HR = 1.61 [1.08–2.40]	p = 0.01 HR = 1.6 [1.11–2.34]

Legends: Multivariate Cox regression analyses were conducted for the whole lung cancer group, and for subgroups including lung cancer with COPD, lung cancer without COPD, squamous cell carcinomas and adenocarcinomas

We adjusted for explanatory variables like packyear, histology, gender, age, and stage

*OS* overall survival, *PFS* progression free survival, *HR* [] Hazard Ratio with 95% confident interval

## Discussion

The main findings in this study of circulating markers in patients with LC and COPD were as follows: 1) Inflammation, endothelial cell activation and ECM remodelling as reflected by these markers, are more pronounced in patients with COPD than in patients with LC, 2) Higher levels of several markers are found in LC patients with COPD than in LC patients without COPD, 3) Higher levels of sTNFR1 and OPG were significantly associated with better survival in the LC group, inversely for CRP and 4) The prognostic impact of circulating markers was different for patients with adenocarcinomas and squamous cell carcinomas and was to some degree influenced by accompanying COPD. Our study underscores the need for including COPD patients as a control group when examining serum markers in patients with LC, and also shows that some of examined proteins (e.g., OPG, sTNFR1 and CRP) could have a potential as prognostic marker in LC patients.

### Circulating levels of markers of inflammation, endothelial cell activation and ECM remodelling in COPD and lung cancer

We observed that inflammation, endothelial cell activation and ECM remodelling as determined by the chosen circulating markers were more prominent in COPD patients than in LC patients. It is known that inflammatory processes may have systemic effects, for instance mirrored by reduced performance status and fever occurring as a consequence of local inflammation. Inflammation may reflect but could also worsen other manifest comorbidities in a patient, like heart disease and diabetes [14]. These effects are modulated by signalling substances like the ones we have studied. It is well known that the same markers can be both tumour promoting and part of tumour defence depending on the context, illustrating the complexity of the involved systems [15]. Chronic inflammatory mediators exert pleiotropic effects in the development of cancer. On the one hand, inflammation favours carcinogenesis; on the other hand, inflammation can stimulate immune effector mechanisms that might limit tumour growth. Patients with COPD are 3–4 times more likely to develop LC than smokers without COPD, and reduced air flow increases the risk significantly [16]. Our finding that some of these markers are present in higher levels in the patients with COPD than in LC patients might be due to a downregulation of inflammation and immune activation by cancer cells. Our findings could also be explained by genetic differences in the ability to increase the level of defence, and hence reflect an increased susceptibility to develop LC. The patients with COPD who do not develop this defence are consequently overrepresented in the cancer patients. A study with serial blood samples, before and after cancer development would elucidate this question.

### Impact on survival

High levels of sTNFR1 were significantly associated with improved overall and progression free survival. In the lungs, TNF and its receptors (TNFRs) are expressed by LC cells [7]. A loss of TNF receptor expression has been demonstrated in advanced LC. Our results are in line with an earlier study showing that higher level of TNFR1 positivity independently predicts favourable outcome in NSCLC, particularly in tumours with no clinically distant metastasis [8]. OPG, also inferring improved prognosis in subgroups, is a soluble cytokine receptor, and a member of the tumour necrosis factor (TNF) receptor superfamily [17]. Interestingly, increased OPG levels did not infer an improved prognosis in adenocarcinomas, but in squamous cell carcinomas. This might be due to limitations in number of patients, but might also reflect biological differences. For instance, squamous cell carcinoma development is more associated with smoking history than development of adenocarcinoma. Some studies have indicated that high levels of OPG are associated with metastatic potential which would be in contrary to our findings [18]. Our study also illustrates the complex role of inflammation and TNF related molecules. High levels of CRP were significantly associated with poor overall survival regardless of COPD status, which is in line with earlier studies [19, 20].

PARC is a chemokine predominantly produced in lungs, and elevated levels of PARC have previously been shown to be associated with hospitalization and mortality in patients with COPD [21]. Our study showed that higher levels of PARC were associated with reduced progression free survival in the total LC group. Interestingly, concomitant COPD did not appear to affect the prognostic value of PARC levels in LC patients.

While sTNFR1 and CRP had prognostic value for both squamous and adenocarcinoma, some proteins showed histology-dependent association with survival. High levels of OPG and low levels of vWF were significantly associated with both better progression free survival and overall survival in squamous carcinoma. Only one study has investigated the vWF antigen levels of NSCLC patients and found that vWF is not substantially altered [22]. A small subset of these patients will have a depletion of circulating vWF antigen, probably because of a paraneoplastic process associated with an advanced stage of disease. CatS was associated with better overall survival in adenocarcinoma, which is in accordance with clinical evidences indicating that up-regulation of CatS in many human cancers is correlated with malignant progression and poor patient prognosis [23].

Studies have shown that PTX3, AXL og ALCAM are associated with metastatic lung cancer. This can explain why these markers were not significantly elevated in lung cancer patients or associated with prognosis in our study. In our material, all three were significantly elevated in the COPD group.

The serum levels of these proteins are affected in several different human situations, and the serum levels in normal humans can vary as a response to different stimuli. This calls for caution in the interpretation.

### Study limitations and considerations

A validation step in an independent similar cohort would strengthen our results. In addition, the biological interpretation of our findings would benefit from analyses of serial serum samples from COPD-patients who later develop LC, comparing serum levels prior to cancer and after a cancer is evident.

In our study, we chose to merge patients with mild COPD with the LC without COPD. There is a known risk of over-staging mild COPD in elderly patients, and merging mild COPD with no COPD is commonly used in elderly [24]. Our LC patients had a median age of 66.7 years, and we classified the patients with mild COPD with the cancer patients without COPD. In our study, there is an unbalance in the severity of COPD, as most of the LC patients with COPD in our study had moderate COPD (86%) while only 38% of the COPD patients had moderate COPD and 43% had severe COPD. Severe COPD is a contraindication for surgical treatment of lung cancer, due to the reduced lung function, meaning that this population is underrepresented in our lung cancer cohort of early stage lung cancer patients. On the contrary, patients with severe COPD are common among the patients followed closely by pulmonologists, and are present in our control population. However, we believe that the signals found in our serum analyses, represent interesting biological characteristics, and should be pursued further.

### Concluding remarks

Our key observations were that the presence of COPD influences circulating inflammation markers in LC patients and the prognostic significance of some proteins depends on the presence of COPD. Furthermore, we identified that chronic inflammation, mirrored by these biomarkers, was more accentuated in COPD patients than in LC patient regardless of their COPD status. This knowledge could have implications for biomarker research in LC screening.

## Additional file

Additional file 1: **Table S1.** Serum protein levels. All data from the serum analyses are presented. This includes results for all proteins and all samples. (XLSX 64 kb)

## Abbreviations

COPD: Chronic obstructive pulmonary disease
COPD^only^: COPD group – no cancer
CT: Computed tomography
ECM: Extracellular matrix
FEV1: Forced expiratory volume in 1 s
FVC: Forced vital capacity
GOLD: The Global Initiative for Chronic Obstructive Lung Disease
LC: Lung cancer
LC^COPD^: Lung cancer with COPD
LC^only^: Lung cancer without COPD
LC^tot^: Lung cancer total - lung cancer with and without COPD
MANCOVA: Multivariate analysis of covariance
NSCLC: Non-small cell lung carcinoma
